# The Roles of Noncoding RNAs in the Development of Osteosarcoma Stem Cells and Potential Therapeutic Targets

**DOI:** 10.3389/fcell.2022.773038

**Published:** 2022-02-16

**Authors:** Jinxin Liu, Guanning Shang

**Affiliations:** Department of Orthopedic Surgery, Shengjing Hospital, China Medical University, Shenyang, China

**Keywords:** noncoding RNA, miRNA, stem cell, osteosarcoma, lncRNAs

## Abstract

Osteosarcoma (OS) is the common bone tumor in children and adolescents. Because of chemotherapy resistance, the OS patients have a poor prognosis. The one reason of chemotherapeutic resistance is the development of cancer stem cells (CSCs). CSCs represent a small portion of tumor cells with the capacity of self-renewal and multipotency, which are associated with tumor initiation, metastasis, recurrence and drug resistance. Recently, noncoding RNAs (ncRNAs) have been reported to critically regulate CSCs. Therefore, in this review article, we described the role of ncRNAs, especially miRNAs, lncRNAs and circRNAs, in regulating CSCs development and potential mechanisms. Specifically, we discussed the role of multiple miRNAs in targeting CSCs, including miR-26a, miR-29b, miR-34a, miR-133a, miR-143, miR-335, miR-382, miR-499a, miR-1247, and let-7days. Moreover, we highlighted the functions of lncRNAs in regulating CSCs in OS, such as B4GALT1-AS1, DANCR, DLX6-AS1, FER1L4, HIF2PUT, LINK-A, MALAT1, SOX2-OT, and THOR. Due to the critical roles of ncRNAs in regulation of OS CSCs, targeting ncRNAs might be a novel strategy for eliminating CSCs for OS therapy.

## Introduction

Osteosarcoma (OS) is the common bone tumor in children and adolescents, which causes a huge healthy problem in childhood (Siegel et al., 2021; Sung et al., 2021). Because a majority of OS patients at diagnosis have micro-metastasis, chemotherapy is often the first strategy for OS treatment (Gill and Gorlick, 2021). However, the drug resistance causes poor outcomes of OS therapy and leads to lower survival rate (Hattinger et al., 2021; Yan and Xiang, 2021). Drug resistance could be due to the development of cancer stem cells (CSCs) in tumorigenesis and progression (Akbar Samadani et al., 2020). CSCs represent a small group of tumor cells with the capacity of self-renewal and multipotency (Izadpanah et al., 2020). It has been documented that CSCs are involved in tumor initiation, metastasis, recurrence and drug resistance. CSCs were identified in a variety of human cancers including OS (Brown et al., 2017; Schiavone et al., 2019). One study identified that CD-117 and Stro-1 might be CSC biomarkers for mouse and human OS, which is involved in tumor metastasis and doxorubicin resistance (Adhikari et al., 2010). Aldehyde dehydrogenase (ALDH) has been considered as a CSC biomarker in OS (Belayneh and Weiss, 2020; Izadpanah et al., 2020). In addition, CD44, CD105, CD199, CD133, CD271, ABCG2, and Sca-1 were repowered as biomarkers for OS CSCs (Yan et al., 2016). Three important pluripotent proteins Sox2, Nanog and Oct3/4 were also correlated with OS CSCs (Yan et al., 2016). Targeting CSCs could be useful for blockade of tumor metastasis and overcoming drug resistance in OS.

## Noncoding RNAs in OS

A number of studies have demonstrated that noncoding RNAs (ncRNAs) are involved in the development, diagnosis, prognosis and treatment of OS (Yang et al., 2021). It has been documented that ncRNAs cannot encode proteins but can regulate gene expression, which include microRNAs (miRNAs), long ncRNAs (lncRNAs) and circRNAs (Slack and Chinnaiyan, 2019). MiRNAs often have 18–25 nucleotides in length and target specific mRNAs via completely or partially complementary binding with 3′UTR of mRNAs (Gebert and Macrae, 2019). LncRNAs with >200 nucleotides exert their functions mainly via sponging miRNAs and targeting specific substrates. Emerging evidence has dissected that ncRNAs participate in OS tumorigenesis and progression (Ghafouri-Fard et al., 2021). For example, ncRNAs are involved in chemotherapeutic drug resistance in osteosarcoma (Ferretti and Leon, 2021; Lin et al., 2021). In recent years, ncRNAs were reported to critically participate in CSCs in a variety of cancers, including OS (Lei et al., 2020; Humphries et al., 2021; Melendez-Zajgla and Maldonado, 2021). Therefore, in the following paragraphs, we will discuss the role of ncRNAs, especially miRNAs, including miR-26a, miR-29b, miR-34a, miR-133a, miR-143, miR-335, miR-382, miR-499a, miR-1247, and let-7days, and lncRNAs, such as B4GALT1-AS1, DANCR, DLX6-AS1, FER1L4, HIF2PUT, LINK-A, MALAT1, SOX2-OT, and THOR, and circRNAs including circ_0001658, circ_0002052 and circPIP5K1A, in regulating CSCs development and potential mechanisms.

## miRNAs Regulate Osteosarcoma CSCs

It is clear that miRNAs participate in regulation of OS CSCs in various types of human cancers, including OS (Table 1). One study determined the genetic characterizations of 3AB-OS CSC line that was established from MG63 cells, and found that 189 differentially expressed miRNAs were existed in 3AB-OS CSCs compared with their parental MG63 cells (Di Fiore et al., 2013). Among these miRNAs, let-7, miR-98 and miR-29a, b, c were downregulated in 3AB-OS CSCs (Di Fiore et al., 2013). One group used DNA microarray and detected the miRNA expression profile in OS cells with CD117 and Stro-1 positive compared with CD117 and Stro-1 negative OS cells (Zhao et al., 2015). This study identified five downregulated miRNAs, including miR-15a, miR-212, miR-302a, miR-423-5p and miR-1247, and three upregulated miRNAs, such as miR-890, miR-518b and miR-1243 (Zhao et al., 2015), suggesting that miRNAs could participate in CSC regulation.

**TABLE 1:** miRNAs regulate CSCs in OS.

miRNAs	Expression	Genes and pathways	References
miR-26a	Down	Jagged-1	Lu et al. (2017)
miR-29b	Down	PI3K/Akt, STAT3	(Di Fiore et al., 2013; Di Fiore et al., 2014; Li et al., 2020b)
miR-34a	Down	DNMT1, Bcl-2	(Liang et al., 2019a; Liang et al., 2019b)
miR-133a	Up	SGMS2, UBA2, SNX30, ANXA2	Fujiwara et al. (2014)
miR-143	Down	KIAA1429, Notch-1	Han et al. (2020)
miR-335	Down	POU5F1	Guo et al. (2017)
miR-382	Down	YB-1	Xu et al. (2015)
miR-499a	Down	SHKBP1	Di Fiore et al. (2016)
miR-1247	Down	MAP3K9	Zhao et al. (2015)
let-7d	Down	CXCR4, MMP-9, VersicanV1, caspase-3, Bcl-2, E-cadherin, N-cadherin, Vimentin, E2F, CCND2, Lin28B, HMGA2	Di Fiore et al. (2016)

## miRNAs Regulate Proliferation of Osteosarcoma CSC Cells

### miR-26a

Evidence has revealed that miR-26a is critically involved in osteosarcoma progression via regulating several downstream targets (Song et al., 2014; Liu et al., 2018). Downregulation of miR-26a was observed and associated with poor prognosis in osteosarcoma patients (Song et al., 2014). For example, miR-26a blocked the migration and invasion of osteosarcoma cells via directly inhibiting HMGA1 (Liu et al., 2018). Similarly, miR-26a retarded the migratory and invasive capacity of osteosarcoma cells via repressing EZH2 expression (Song et al., 2014). Tan et al. reported that miR-26a attenuated cell proliferation via inhibiting IGF-1 expression in osteosarcoma cells (Tan et al., 2015). Li et al. found that miR-26a could reverse doxorubicin resistance via inhibiting MCL1 in osteosarcoma cells (Li and Ma, 2021). Surprisingly, one study reported that miR-26a might be an oncogene in osteosarcoma. Qu et al. found that miR-26a enhanced cell growth and tumor metastasis via regulating the Wnt/β-catenin pathway by inhibiting GSK-3β in osteosarcoma (Qu et al., 2016). Another study showed that miR-26a repressed stem cell-like properties via inhibition of Jagged-1 in osteosarcoma (Lu et al., 2017). Notably, decreased expression of miR-26a was linked to lung metastasis and poor survival in patients with osteosarcoma (Lu et al., 2017). Moreover, lower expression of miR-26a existed in osteosarcoma CSCs, and lentivirus-mediated upregulation of miR-26a reduced the expression of stem cell biomarkers, including SOX2, CD133, OCT3/4, Nanog, and nucleostemin in osteosarcoma cells (Lu et al., 2017). ZOS and 143B cells formed smaller and fewer sarcosphere and had a reduction of the ALDH activity after infection with lentiviruses carrying miR-26a. Moreover, miR-26a suppressed the expression of Jagged-1, and led to inhibition of tumor cell growth *in vitro* and *in vivo* (Lu et al., 2017).

### miR-34a

Zou et al. revealed that miR-34a expression was lower in osteosarcoma stem-like cells, and overexpression of miR-34a reduced the expression of the stem cell markers and retarded the osteosphere formation (Zou et al., 2017). Zhang et al. discovered that miR-34a worked as a suppressor in regulation of osteosarcoma dedifferentiation into CSCs via inhibition of Sox2 (Zhang et al., 2018). Liang et al. reported that miR-34a was increased after DNMT1 downregulation, leading to suppression of stemness markers expression, including CD133, CD44, Oct4, Sox2, Bmi1 and ABCG2 in osteosarcoma stem-like cells (Liang et al., 2019b). Consistently, overexpression of DNMT1 reduced the expression of miR-34a and promoted the expression of stemness markers in osteosarcoma stem-like cells (Liang et al., 2019b). Moreover, Liang et al. found that isovitexin, a natural flavonoid, reduced the expression of CD133, CD44, ALDH1 and ABCG2 at mRNA levels in osteosarcoma sphere cells, leading to suppression of tumor growth and induction of apoptosis. Mechanistic study showed that isovitexin reduced DNMT1 expression and activity, upregulated miR-34a and attenuated the expression of Bcl-2 in osteosarcoma sphere cells (Liang et al., 2019a).

### miR-143

Evidence demonstrated that miR-143 is linked to the survival of OS cells with ALDH1+CD133+ and participated in drug resistance (Zhou et al., 2015). Loss of miR-143 expression was associated with poor survival of OS patients. Overexpression of miR-143 overcame drug resistance via inhibition of ATG2B, LC3-1 and Bcl-2 in U2OS- and SaOS-2-resistant cells (Zhou et al., 2015). In addition, miR-143-3p is involved in osteosarcoma development and progression. MiR-143-3p was identified as a potential marker for diagnosis and prognosis in osteosarcoma patients, because low expression of miR-143-3p was correlated with tumor size, stage and metastasis (Yang et al., 2020). Sun et al. showed that miR-143-3p repressed cell proliferation and invasion via suppression of FOSL2 in osteosarcoma (Sun et al., 2018). Hou et al. observed that miR-143-3p reduced cell growth, migratory and invasive capacity via attenuation of MAPK7 expression in osteosarcoma (Hou et al., 2019). Han et al. uncovered that ectopic expression of miR-143-3p blocked stemness features in osteosarcoma cells, including CD44, Oct4, Nanog and Notch1 (Han et al., 2020). Moreover, miR-143-3p inhibited KIAA1429 expression via binding with its 3′-UTR in U2OS and 143B cells (Han et al., 2020). Furthermore, miR-143-3p suppressed proliferation and invasiveness in a KIAA1429-dependent manner in osteosarcoma cells (Han et al., 2020).

### miR-1247

Wei et al. reported that miR-1247 repressed cell viability and blocked tumor metastasis via inhibiting NRP1 expression and mediating Wnt/β-catenin pathway in OS (Wei et al., 2019). Evidence showed that miR-1247 was downregulated in OS cells with CD117+Stro-1+ (Zhao et al., 2015). Moreover, miR-1247 bound to MAP3K9 and inhibited its expression in OS cells. MAP3K9 facilitated proliferation of OS cells and stem cell sphere formation in CD117+Stro-1+ cells (Zhao et al., 2015). Restoration of miR-1247 reduced the clonogenic growth and suppressed tumor spheres in OS cells (Zhao et al., 2015). This study implied that miR-1247 could be involved in regulation of the self-renewal of OS CSCs.

## miRNAs Regulate Metastasis of Osteosarcoma CSC Cells

### miR-133a

One investigation revealed the association between miR-133a expression and osteosarcoma-initiating cells (Fujiwara et al., 2014). This study found that 20 miRNAs were upregulated in CD133 (high) OS cells, including miR-133a (Fujiwara et al., 2014). Moreover, miR-133a promoted cell invasion in CD133 (high) OS cells. Moreover, OS patients with poor prognosis have a high expression of miR-133a (Fujiwara et al., 2014). Chemotherapeutic treatment increased the expression of miR-133a in OS cells. Furthermore, miR-133a exerted its functions in part via inhibition of SGMS2, UBA2, SNX30 and ANXA2 in OS cells (Fujiwara et al., 2014). Therefore, miR-133a could be involved in development and metastasis of OS CSCs.

### miR-382

miR-382 has been reported to regulate tumor growth and metastasis in osteosarcoma cells (Xu et al., 2014). Osteosarcoma patients with lower expression of miR-382 had a poor chemoresponse and poor survival (Xu et al., 2014). Ectopic expression of miR-382 reduced growth and chemoresistance of osteosarcoma cells via attenuating KLF12 and HIPK3 (Xu et al., 2014). miR-382-5p, a miRNA species of miR-382, was reported to govern hematopoietic stem cell differentiation via the inhibition of MXD1 (Zini et al., 2016). One study revealed that miR-382 was involved in regulation of CSC populations in colorectal cancer spheroid cells (Rengganaten et al., 2020). miR-382 reduced metastasis and relapse via suppression of YB-1 in osteosarcoma cells (Xu et al., 2015). The upregulation of miR-382 repressed EMT and lung metastasis as well as reduced the population of CSCs (CD133 high) in LM-5 and M132 osteosarcoma cells (Xu et al., 2015). In keeping with this result, knockdown of miR-382 induced EMT and metastasis and elevated the percentage of CSCs in SaOs-2 and HuO9 osteosarcoma cells (Xu et al., 2015). Consistently, the numbers of ALDH1-positive cells were changed after miR-382 modulation. Notably, miR-382 retarded the capacity of osteosarcoma cells to form osteospheres (Xu et al., 2015). The clinical data demonstrated that lower expression of miR-382 was existed in highly metastatic osteosarcoma cells and relapsed osteosarcoma specimens. Moreover, miR-382 expression level was linked to relapse and survival in patients with osteosarcoma. Furthermore, ectopic expression of miR-382 blocked CSC-mediated tumor formation in mice (Xu et al., 2015). Notably, miR-382 in combination with doxorubicin blocked disease relapse in osteosarcoma in nude mice. Mechanistically, miR-382 controlled EMT, stemness and tumor metastasis and relapse via inhibiting YB-1 (Xu et al., 2015).

## miRNAs Regulate Drug Resistance of Osteosarcoma CSC Cells

### miR-29b-1

Zhang et al. observed that miR-29b-1 decreased proliferation and migration of OS cells via blocking the expression of VEGF (Zhang et al., 2014). Zhu et al. found that miR-29b exerted antitumor activity in OS via modulation of CDK6 (Zhu et al., 2016). Xu and others reported that miR-29 family targeted COL3A1 and Mcl-1 and increased methotrexate sensitivity in OS cells (Xu et al., 2018). Moreover, miR-29b was reported to sensitize OS cells to doxorubicin via suppressing MMP-9 (Luo et al., 2019). Overexpression of miR-29b increased the radiosensitivity of OS cells via targeting PTEN/Akt/Sp1 pathway (Kim et al., 2020). One research dissected that miR-29b-1 repressed proliferation, self-renewal and overcame chemoresistance in 3AB-OS CSCs (Di Fiore et al., 2014). miR-29b expression was downregulated in 3AB-OS CSCs (Di Fiore et al., 2013), and overexpression of miR-29b-1 impaired proliferation, sarcosphere ability and colony formation ability (Di Fiore et al., 2014). Upregulation of miR-29b-1 sensitized chemotherapeutic drug efficacy in 3AB-OS cells. Moreover, miR-29b-1 reduced stemness properties via suppression of Oct3/4, Sox2 and Nanog in 3AB-OS CSCs (Di Fiore et al., 2014). Recently, soft substrate inhibited miR-29b expression and upregulated Spin one expression, leading to activation of PI3K/Akt and STAT3 pathways, which resulted in self-renewal, differentiation and drug resistance in OS cells (Li S. et al., 2020).

### miR-335

Emerging evidence has revealed that miR-335 overexpression retarded invasion and migration of osteosarcoma cells via repressing ROCK1 and SNIP1 expression (Wang et al., 2013; Xie et al., 2019). LncRNA TUG1 enhanced invasion and migration of OS cells via sponging miR-335-5p and upregulating ROCK1 (Wang et al., 2017b). LncRNA DANCR enhanced ROCK-1-induced proliferation and motility of OS cells via sponging miR-335-5p and miR-1972 (Wang et al., 2018). LncRNA LOC100129620 increased proliferation and migration via interacting with miR-335-3p and regulating CDK6 in OS cells (Chen et al., 2021). LncRNA CRNDE elevated OS progression by suppression of miR-335-3p (Yu et al., 2021). Upregulation of miR-335 induced apoptosis and attenuated cell viability via inhibition of survivin expression in OS cells (Liu et al., 2016). Clinically, the expression of miR-335 was lower in OS tissues compared with normal control tissues (Wang et al., 2013; Liu et al., 2016). Notably, lower expression of miR-335 was positively correlated with advanced clinical stage and distant metastasis (Wang et al., 2017a). Guo et al. reported that the downregulation of miR-335 was reported in osteosarcoma stem cells (Guo et al., 2017). Furthermore, miR-335 inhibited stem cell-like properties via suppression of POU5F1 in osteosarcoma (Guo et al., 2017). miR-335 upregulation inhibited the expression of CD117, Stro-1, and Sox2 in osteosarcoma cells. Inhibition of miR-335 enhanced stem cell-like properties and increased invasion of osteosarcoma cells. Moreover, miR-335 overexpression increased sensitivity of cisplatin in osteosarcoma cells (Guo et al., 2017).

### miR-499a

Wang et al. reported that TGF-β-triggered EMT reduced the expression of miR-499a because Snail1 and Zeb1 bound with miR-499a promoter (Wang et al., 2019). Upregulation of miR-499a reduced TGF-β-mediated erlotinib resistance via inhibition of SHKBP1 in CD166 + OS CSCs (Di Fiore et al., 2016). The high ratio of the SHKBP1 and miR-499a were associated with EMT and erlotinib resistance in OS samples (Di Fiore et al., 2016). This study indicated that miR-499a might be involved in drug resistance in OS CSCs.

### Let-7d

One research showed that the expression of let-7days miRNA was decreased in the 3AB-OS CSCs (Di Fiore et al., 2013). Upregulation of let-7d inhibited cell proliferation via suppressing the expression of CCND2 and E2F2 and upregulating p21 and p27 expression in 3AB-OS CSCs (Di Fiore et al., 2016). Moreover, overexpression of let-7d reduced sarcosphere capacity and attenuated the expression of several stem markers, including Sox2, Lin28B, Oct3/4, Nanog, and HMGA2, in 3AB-OS CSCs (Di Fiore et al., 2016). Furthermore, overexpression of let-7d reduced vimentin expression and N-cadherin expression, but increased E-cadherin expression, leading to mesenchymal to epithelial transition (Di Fiore et al., 2016). Interestingly, overexpression of let-7days elevated the expression of CXCR4, MMP-9 and VersicanV1, resulting in promotion of migration and invasion in 3AB-OS CSCs (Di Fiore et al., 2016). Moreover, upregulation of let-7d increased resistance to chemotherapy drugs, which was associated with downregulation of caspase-3 and upregulation of Bcl-2 expression in 3AB-OS CSCs (Di Fiore et al., 2016).

## LncRNAs Regulate Osteosarcoma CSCs

Recently, multiple studies have suggested that lncRNAs could regulate properties of osteosarcoma. For example, lncRNA GClnc1 enhanced tumorigenesis via suppression of p53 signaling pathway in osteosarcoma (Sui et al., 2018). Knockdown of lncRNA 91H blocked the tumorigenesis via induction of methylation of CDK4 promoter in osteosarcoma (Cheng et al., 2021). Overexpression of lncRNA FGFR3-AS1 facilitated osteosarcoma growth via governing the antisense transcript FGFR3 (Sun et al., 2016). Several studies have revealed that lncRNA HOTTIP increased cell proliferation, migration, invasion, EMT, chemoresistance, in osteosarcoma (Li Z. et al., 2016; Tang and Ji, 2019; Liu et al., 2020; Yao et al., 2021). LncRNA HULC promoted the progression of osteosarcoma via targeting the miR-372-3p/HMGB1 (Li Y. et al., 2020). In addition, inhibition of lncRNA UCA1 reduced tumorigenesis and metastasis via targeting miR-513b-5p/E2F5 axis and CREB1-mediated EMT and PI3K/AKT/mTOR axis in osteosarcoma (Ma H. et al., 2019; Zhang et al., 2021). Here, we will briefly describe the functions and molecular insights of these lncRNAs in governing CSC features in osteosarcoma (Table 2).

**TABLE 2:** LncRNAs regulate CSCs in OS.

LncRNAs	Expression	Genes and pathways	References
B4GALT1-AS1	Up	YAP	Li et al. (2018)
DANCR	Up	miR-33a-5p, AXL, PI3K/Akt	Jiang et al. (2017)
DLX6-AS1	Up	miR-129-5p, DLK1, Wnt	Zhang et al. (2018)
FER1L4	Down	PI3K/Akt	Ma et al. (2019b)
HIF2PUT	Down	HIF-2α	(Wang et al., 2015; Li et al., 2016a)
LINK-A	Up	TGF-β1	Kong et al. (2020)
MALAT1	Up	miR-129-5p, RET-Akt, PI3K	Chen et al. (2018a)
SOX2-OT	Up	SOX2	Wang et al. (2017d)
THOR	Up	SOX9	Wu et al. (2019b)

## LncRNAs Regulate Proliferation of Osteosarcoma CSC Cells

### lncRNA DANCR

Recently, several studies identified that DANCR was increased in osteosarcoma tissues and tumor cell lines. High expression of DANCR was linked to tissue typing and TNM stage as well as metastasis in osteosarcoma patients (Jiang et al., 2017; Wang et al., 2018; Zhang W. et al., 2020). One study revealed that deficient of DANCR suppressed growth and autophagy, and triggered apoptosis via sponging miR-216a-5p and increasing the expression of SOX5 (Pan et al., 2020). Moreover, DANCR inhibited migration and invasion via targeting miR-149 and its downstream MSI2 in osteosarcoma (Zhang W. et al., 2020). In addition, DANCR acted as a ceRNA to sponge miR-335-5p and miR-1972, leading to inhibition of ROCK1 expression and depression of proliferation and motility of osteosarcoma cells (Wang et al., 2018). Jiang et al. reported that DANCR decoyed miR-33a-5p and upregulated AXL expression, contributing to tumor growth, migration, invasion and lung metastasis (Jiang et al., 2017). Mechanistically, DANCR stimulated tumor malignant phenotype via enhancement of CSCs features, which might be due to activation of PI3K/Akt signaling pathway in osteosarcoma (Jiang et al., 2017). Yuan et al. reported that DANCR promoted cell stemness property via derepressing CTNNB1 in hepatocellular carcinoma (HCC) cells (Yuan et al., 2016). DANCR expression was high in HCC cells with stem-like features (Yuan et al., 2016). DANCR depletion suppressed expression of several CSC markers, such as CD44, ABCG2, and ALDH1 in TNBC cells (Sha et al., 2017). In lung cancer cells, DANCR overexpression increased cell stemness via decoying miR-216a expression and subsequent activation of Wnt/β-catenin axis (Yu et al., 2020).

### LncRNA DLX6-AS1

LncRNA DLX6-AS1 deficiency blocked tumor development due to inhibition of CADM1 promoter methylation and suppression of STAT3 pathway in liver CSCs (Wu D.-M. et al., 2019). Knockdown of DLX6-AS1 repressed spheroid formation and suppressed the expression of stem markers in liver CSCs, such as SOX2, Nanog, OCT4, CD13 and CD133 (Wu D.-M. et al., 2019). Similarly, DLX6-AS1 overexpression promoted stemness of osteosarcoma cells via interacting with miR-129-5p and activation of DLK1, leading to activating Wnt pathway (Zhang et al., 2018). Patients with high expression of DLX6-AS1 often had poor grade, advanced stage and poor overall survival (Zhang et al., 2018). Deficient of DLX6-AS1 decreased sphere size and number, and CD117+Stro-1+ cells were decreased in osteosarcoma cells after DLX6-AS1 silencing (Zhang et al., 2018).

### LncRNA HIF2PUT

LncRNA HIF2PUT was reported to regulate the proliferation, invasion and migration in osteosarcoma cells (Zhao D. et al., 2019). One group reported that HIF2PUT overexpression attenuated cell growth and motility in U2OS and MG-63 cells (Zhao D. et al., 2019). HIF2PUT expression was correlated with clinical features of osteosarcoma patients, including tumor size, stage, distant metastasis, and OS and DFS (Li W. et al., 2016). Interestingly, HIF2PUT was also reported to be highly expressed in osteosarcoma, suggesting that the deeper investigation is necessary to dissect the role of HIF2PUT in osteosarcoma tumorigenesis. HIF2PUT has been revealed to control CSCs in several types of human malignancies. For instance, HIF2PUT overexpression repressed CSC properties via targeting HIF-2α in colon cancer (Yao et al., 2015). Downregulation of HIF2PUT decreased the expression of stemness biomarkers in colon cancer DLD-1 and HT29 cells, leading to blockade of spheroid formation (Yao et al., 2015). However, downregulation of HIF2PUT elevated cell growth and migratory ability in MG63 cells, and elevation of HIF2PUT showed an opposite effect in osteosarcoma cells (Wang et al., 2015). Moreover, increased HIF2PUT led to a reduction of CD133 + MG63 cells and inhibition of sphere-forming ability, while decreased HIF2PUT resulted in an induction of CD133 positive cells and promotion of sphere-forming capacity (Wang et al., 2015). Another study validated that HIF2PUT upregulation inhibited sphere formation of osteosarcoma cells (Zhao D. et al., 2019). A mechanistical experiment showed that HIF2PUT could target HIF-2α expression and perform its biological function in osteosarcoma cells (Wang et al., 2015). Furthermore, the clinical data revealed that HIF2PUT expression was linked to HIF-2α levels in tumor tissues of osteosarcoma patients (Wang et al., 2015; Li W. et al., 2016).

### LncRNA THOR

LncRNA THOR was found to involve in CSC maintenance in TNBC cells (Wang B. et al., 2020). THOR expression was higher in TNBC tissues than that in luminal A-type and luminal B-type breast cancer (Wang B. et al., 2020). Silencing of lncRNA THOR attenuated the expression of stemness regulatory factors, including CD44, Nanog, and Oct4, and reduced ALDH1 activity, leading to suppressing the sphere-formation ability, which is evidenced by reduced sphere size and number in MDA-MB-231 and MDA-MB-453 cells (Wang B. et al., 2020). Overexpression of THOR promoted CSC properties and increased stemness factor expression, indicating that THOR might promote stemness of TNBC cells. Moreover, THOR interacted with β-catenin mRNA and increased its mRNA stability and elevated its expression (Wang B. et al., 2020). In line with the role of THOR in regulating CSCs, Cheng et al. found that THOR promoted CSC expansion and stimulated the self-renewal ability via targeting β-catenin axis in hepatocellular carcinoma (Cheng et al., 2019). Similarly, THOR knockdown reduced the stemness via inhibition of multiple stemness markers, such as CD44, SOX2, SOX9, Nanog, Oct1/2/4, and ALDH, in MKN-45 and BGC-23 gastric cancer cells (Song et al., 2018). Silencing of THOR attenuated the spheroids size and number and reduced the ability of spheroid formation in gastric cancer (Song et al., 2018). Moreover, depletion of THOR reduced SOX9 expression via binding to and increasing SOX9 mRNA stability (Song et al., 2018). In nasopharyngeal carcinoma (NPC) cells, THOR decreased sensitivity of cisplatin via promoting CSC stemness (Gao et al., 2018). THOR interacted with YAP and blocked its translocation to cytoplasm from nuclear, leading to enhancement of YAP transcription activity in NPC cells (Gao et al., 2018). In osteosarcoma cells, THOR increased stemness and migratory capacity via increasing stability of SOX9 mRNA (Wu H. et al., 2019). THOR expression was higher in cell spheroids than that in adherent cells in osteosarcoma. Upregulation of THOR elevated the ALDH activity and enhanced spheroid formation in adherent cells of osteosarcoma, whereas downregulation of THOR showed an opposite function in spheroids (Wu H. et al., 2019). THOR promoted osteosarcoma CSC stemness via increasing SOX9 mRNA stability and upregulating its expression (Wu H. et al., 2019). Altogether, lncRNA THOR participate in controlling CSCs in osteosarcoma.

## LncRNAs Regulate Metastasis of Osteosarcoma CSC Cells

### LncRNA FER1L4

LncRNA FER1L4 plays an anti-tumor role in osteosarcoma development (Fei et al., 2018). The evidence is that lncRNA FER1L4 has a lower expression in tissues of osteosarcoma patients, which is linked to stage and metastasis (Chen ZX. et al., 2018; Ye et al., 2019). In addition, FER1L4 retarded osteosarcoma tumorigenesis via sponging miR-18a-5p and increasing PTEN expression (Fei et al., 2018). FER1L4 was reported to control PDLSCs under compressive stress (Huang et al., 2019). One study found that 72 lncRNAs were increased and 18 lncRNAs were decreased in PDLSCs after static compressive stress (Huang et al., 2019). These lncRNAs contained FER1L4, NEAT1, LUCAT1 and HIF1A-AS2 (Huang et al., 2019). Moreover, FER1L4 stimulated osteogenic differentiation of PDLSCs via binding with miR-874-3p and targeting VEGFA, suggesting that FER1L4 could enhance bone formation (Huang et al., 2020). Furthermore, FER1L4 triggered the autophagy via regulating Akt/FOXO3 signaling pathway in PDLSCs under orthodontic compressive strain (Huang et al., 2021). Ma et al. reported that ectopic expression of FER1L4 inhibited proliferation, induced apoptosis, blocked migration and invasion, suppressed EMT in osteosarcoma cells (Ma L. et al., 2019). Moreover, silencing of FER1L4 upregulated the expression of several stemness biomarkers, including CD44, Oct4, SOX9, Nanog, and ALDH1 (Ma L. et al., 2019). In mechanism, FER1L4 suppressed tumor progression via targeting PI3K/Akt pathway in osteosarcoma.

### LncRNA LINK-A

One group showed that metastatic osteosarcoma patients had a higher level of LINK-A in plasma (Zhao B. et al., 2019). Elevation of LINK-A enlarged migratory and invasive capacity of osteosarcoma cells via induction of HIF-1α expression (Zhao B. et al., 2019). Another group also observed that LINK-A in plasma was highly expressed in osteosarcoma patients (Kong et al., 2020). LINK-A upregulation increased TGF-β1 expression in osteosarcoma cells. Deficient of LINK-A led to suppression of migration and invasion of osteosarcoma cells. Moreover, depletion of LINK-A decreased the percentage of CD133 + cells in osteosarcoma cell lines (Kong et al., 2020). This finding indicated that LINK-A might participate in governing stemness of osteosarcoma.

### LncRNA MALAT1

LncRNA MALAT1 has been discovered to regulate stem cell expression in osteosarcoma (Chen Y. et al., 2018). Chen et al. reported that MALAT1 expression level was increased in tumor tissues and linked to tumor size, metastasis and poor survival in osteosarcoma patients (Chen Y. et al., 2018). Ectopic expression of MALAT1 increased proliferation, migratory and invasive ability in osteosarcoma cells and promoted tumor growth in mice via sponging miR-129-5p and regulating the RET-Akt pathway (Chen Y. et al., 2018). It has been known that CD90, SOX2 and CD133 are well-characterized stemness markers. Moreover, MALAT1 upregulation elevated the expression of CD90, SOX2 and CD133 in SW1353 and SOSP-9607 cells. In consistent, depletion of MALAT1 reduced the expression of CD90, CD133 and SOX2 in osteosarcoma cells (Chen Y. et al., 2018). In line with this finding, MALAT1 overexpression in SW1353 and SOSP-9607 cells resulted in enhancement of CD133 + CD44 ^+^ cell proportion, while depletion of MALAT1 displayed the opposite effects (Chen Y. et al., 2018). This study suggested that MALAT1 enhanced stem cell-like features via promotion of RET expression via targeting miR-129-5p and subsequently activating the PI3K-Akt pathway in osteosarcoma. MALAT1 was highly expressed in patients with osteosarcoma (Wang et al., 2017c). MALAT1 promoted proliferation and metastasis via sponging miR-144-3p and blocking ROCK1/ROCK2 axis in osteosarcoma cells (Wang et al., 2017c).

### LncRNA SOX2-OT

LncRNA SOX2-OT has been characterized as an oncogene and is highly expressed in various cancers (Li PY. et al., 2020). Higher level of lncRNA SOX2-OT was existed in several osteosarcoma cell lines and tumor specimens. Notably, osteosarcoma patients with high level of lncRNA SOX2-OT often have bigger tumor size, advanced stage, high grade and metastasis and poor OS (Wang Z. et al., 2017). An *in vitro* experiment showed that knockdown of lncRNA SOX2-OT attenuated proliferation and migration and invasion of U2OS cells. In consistent, elevation of lncRNA SOX2-OT enhanced proliferation and facilitated invasive and migratory capacity in SaOS-2 cells (Wang Z. et al., 2017). Moreover, SOX2 was confirmed as a downstream target of lncRNA SOX2-OT in osteosarcoma. Strikingly, the expression of stemness biomarkers was downregulated in osteosarcoma cells after lncRNA SOX2-OT knockdown, including ALDH1, Nanog, Oct4, CD44 and CD133 (Wang Z. et al., 2017). Taken together, lncRNA SOX2-OT might regulate CSCs via positively regulating SOX2 in osteosarcoma.

## LncRNAs Regulate Drug Resistance of Osteosarcoma CSC Cells

### LncRNA B4GALT1-AS1

B4GALT1-AS1 has been reported to serve as a ceRNA to sequester the expression of miR-30e, resulting in the upregulation of SOX9 in NSCLC (Lin et al., 2020). B4GALT1-AS1 had an increased expression in NSCLC tissues and cells. Silencing of B4GALT1-AS1 blocked malignant phenotype in A549 and H1299 cells, including cell viability and colony-forming ability (Lin et al., 2020). Deficient of B4GALT1-AS1 reduced clone formation capacity in colon cancer cells, and attenuated the expression of the stemness biomarkers. B4GALT1-AS1 silencing also reduced ALDH1 activity and retarded spheroid formation in colon cancer cells (Wu D.-M. et al., 2019). Mechanistically, B4GALT1-AS1 might enhance the relocation of YAP into nucleus from cytoplasm and promote its transcription, leading to maintenance of CSCs in colon cancer (Wu D.-M. et al., 2019). Similarly, higher expression of B4GALT1-AS1 was observed in osteosarcoma tissues. Depletion of B4GALT1-AS1 decreased proliferation and migratory capacity of osteosarcoma cells, blocked EMT progression, evidenced by an increase of E-cadherin and a decrease of vimentin (Li et al., 2018). Knockdown of B4GALT1-AS1 attenuated the expression of Nanog and ALDH1 and reduced the capability of spheroid formation, suggesting that B4GALT1-AS1 is involved in regulation of osteosarcoma cell stemness (Li et al., 2018). *In vivo* data further confirmed that B4GALT1-AS1 silencing decreased tumor formation in mice. B4GALT1-AS1 promoted the translocation of HuR into cytoplasm from nuclear and led to upregulation of YAP transcription in osteosarcoma (Li et al., 2018). Notably, deficient of B4GALT1-AS1 reduced adriamycin resistance in a YAP-dependent manner in osteosarcoma cells. This study revealed that B4GALT1-AS1 shed light on the regulation of CSC features in osteosarcoma.

## CircRNAs Regulate Osteosarcoma CSCs

Increasing evidence suggests that circRNAs play a pivotal role in osteosarcoma development and progression (Li et al., 2021). Wang et al. found that circ_0001658 increased cell proliferation and tumor metastasis via sponging miR-382-5p and increasing YB-1 axis in osteosarcoma cells (Wang L. et al., 2020). One group identified that miR-382 knockdown triggered EMT and promoted metastasis and increased the percentage of CSCs via suppressing YB-1 in osteosarcoma cells (Xu et al., 2015). Therefore, circ_0001658 could regulate CSCs and osteospheres via targeting miR-382-5p/YB-1 axis in osteosarcoma. Moreover, circ_0002052 knockdown inhibited cell growth, migration and invasion via sponging miR-382 in osteosarcoma, indicating that circ_0002052 might increase CSCs via inhibiting miR-382 (Zhang P.-r. et al., 2020). CircNRIP1 encapsulated by BMSC-EVs aggravated osteosarcoma via targeting miR-532-3p and PI3K/AKT axis (Shi Z. et al., 2021). Shi et al. reported that circPIP5K1A depletion reduced the sphere formation abilities in osteosarcoma cells and decreased the CD133 + CD44 ^+^ cell population (Shi P. et al., 2021). Knockdown of circPIP5K1A reduced the expression of Nanog ad ALDH1 in osteosarcoma cells. Moreover, circPIP5K1A increased YAP expression via regulating miR-515-5p, and miR-515-5p suppressed cancer stemness in osteosarcoma cells (Shi P. et al., 2021). Notably, circPIP5K1A depletion or miR-515-5p mimic inhibited the CSC properties in osteosarcoma cells, suggesting that circPIP5K1A can control CSCs in osteosarcoma (Shi P. et al., 2021).

## ncRNAs in Chondrosarcoma

It is necessary to mention that ncRNAs have been uncovered to play an essential role in another primary bone sarcomas chondrosarcoma, including miR-30a, miR-125b, miR-126, miR-129-5p, miR-145, miR-181a, miR-150, miR-494, and miR-497 (Chang et al., 2015; Li et al., 2015; Lu et al., 2016; Pu et al., 2016; Palmini et al., 2017; Zhang et al., 2017). Several lncRNAs, such as SNHG6 (Pu et al., 2021), RAMP2-AS1 (Cheng et al., 2020), BCAR4 (Shui et al., 2017) and HOTAIR (Bao et al., 2017), have been reported to promote chondrosarcoma development and progression. However, the role of ncRNAs in regulation of chondrosarcoma CSCs is rarely investigated. One report showed that miR-34a in combination with carbon ions irradiation can control chondrosarcoma CSCs (Vares et al., 2020). Therefore, further studies are warranted to determine the functions of ncRNAs in governing chondrosarcoma CSCs.

## Conclusion and Perspectives

**FIGURE 1:**
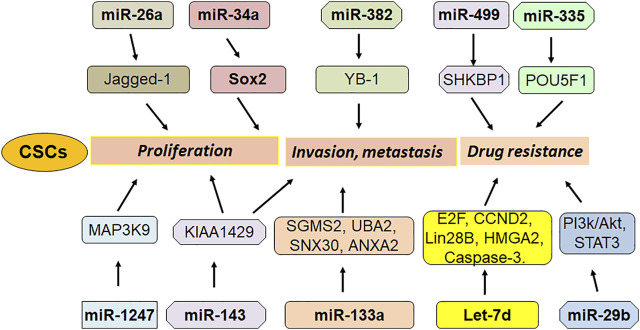
The role of miRNAs in regulation of OS stem cells.

**FIGURE 2:**
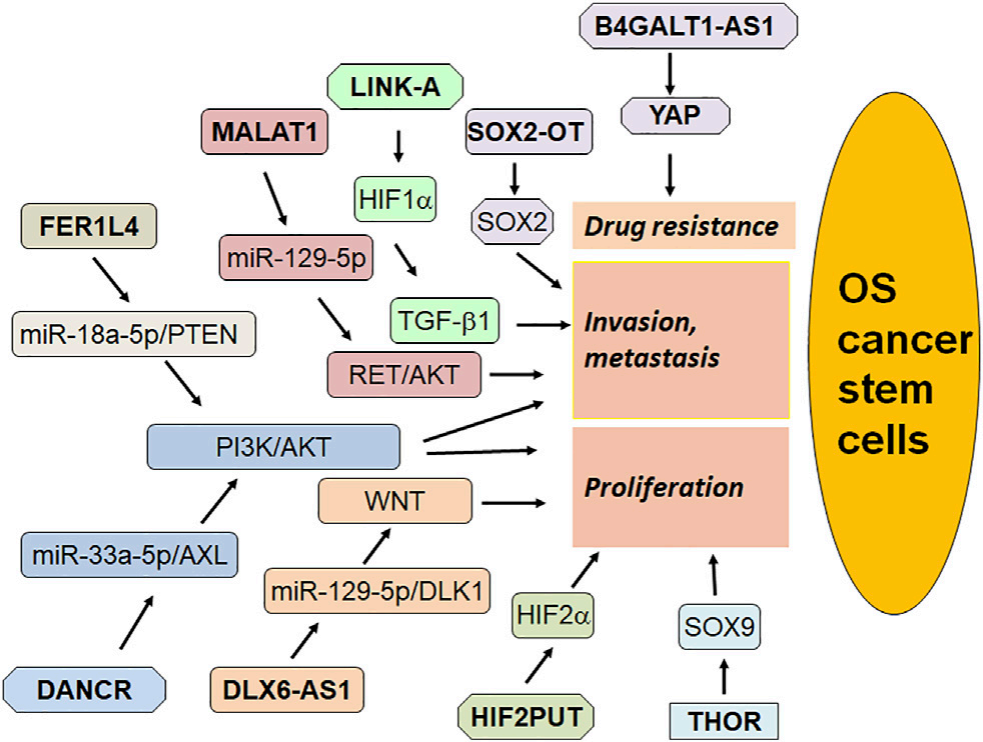
The role of lncRNAs in regulation of OS stem cells.

In conclusion, ncRNAs critically regulate CSCs via different mechanisms in osteosarcoma (Figures 1, 2). Because CSCs are important in tumor initiation, reoccurrence, metastasis and drug resistance, modulating ncRNAs could be helpful for overcoming tumor progression and enhancing drug sensitivity via killing CSCs in osteosarcoma. It is important to mention that numerous ncRNAs are involved in regulating osteosarcoma CSCs. Whether ncRNAs are the most important factors to control CSCs compared with other transcript factors that were involved in CSCs? Among these ncRNAs, which ncRNA is most important factor to govern CSCs in osteosarcoma. It is known that ncRNAs have multiple downstream targets. How can we judge the key targets of ncRNAs in regulating CSCs? Answering these questions will provide the evidence for targeting CSCs via modulation of ncRNAs for osteosarcoma treatment. In addition, it is critical to discover a standardized approach to measure the ncRNAs expression. A useful and ideal deliver system to send ncRNAs to specific organs *in vivo* is also important to establish. LncRNAs have been evaluated for targeting critical cancer-associated genes and they are in different phases of clinical trials (Slaby, 2016). Since discover of possible biomarkers is important for diagnosis and treatment of osteosarcoma, it is essential to determine whether these ncRNAs could be potential biomarkers for detection of osteosarcoma CSCs. Lastly, further investigations are needed to validate whether targeting ncRNAs could control OS CSCs and overcome drug resistance in clinical management of OS in the future.

## References

[B1] Adhikari A. S. Agarwal N. Wood B. M. Porretta C. Ruiz B. Pochampally R. R. (2010). CD117 and Stro-1 Identify Osteosarcoma Tumor-Initiating Cells Associated with Metastasis and Drug Resistance. Cancer Res. 70, 4602–4612. 10.1158/0008-5472.can-09-3463 20460510 PMC3139225

[B2] Akbar Samadani A. Keymoradzdeh A. Shams S. Soleymanpour A. Elham Norollahi S. Vahidi S. (2020). Mechanisms of Cancer Stem Cell Therapy. Clinica Chim. Acta 510, 581–592. 10.1016/j.cca.2020.08.016 32791136

[B3] Bao X. Ren T. Huang Y. Sun K. Wang S. Liu K. (2017). Knockdown of Long Non-coding RNA HOTAIR Increases miR-454-3p by Targeting Stat3 and Atg12 to Inhibit Chondrosarcoma Growth. Cell Death Dis 8–e2605. 10.1038/cddis.2017.31 PMC5386479 28182000

[B4] Belayneh R. Weiss K. (2020). The Role of ALDH in the Metastatic Potential of Osteosarcoma Cells and Potential ALDH Targets. Adv. Exp. Med. Biol. 1258, 157–166. 10.1007/978-3-030-43085-6_10 32767240

[B5] Brown H. K. Tellez-Gabriel M. Heymann D. (2017). Cancer Stem Cells in Osteosarcoma. Cancer Lett. 386, 189–195. 10.1016/j.canlet.2016.11.019 27894960

[B6] Chang L. Shrestha S. Lachaud G. Scott M. A. James A. W. (2015). Review of microRNA in Osteosarcoma and Chondrosarcoma. Med. Oncol. 32, 613. 10.1007/s12032-015-0613-z 25920607

[B7] Chen Y. Huang W. Sun W. Zheng B. Wang C. Luo Z. (2018a). LncRNA MALAT1 Promotes Cancer Metastasis in Osteosarcoma via Activation of the PI3K-Akt Signaling Pathway. Cell Physiol Biochem 51, 1313–1326. 10.1159/000495550 30481748

[B8] Chen Y. Tang G. Qian H. Chen J. Cheng B. Zhou C. (2021). LncRNA LOC100129620 Promotes Osteosarcoma Progression through Regulating CDK6 Expression, Tumor Angiogenesis, and Macrophage Polarization. Aging 13, 14258–14276. 10.18632/aging.203042 34015762 PMC8202873

[B9] Chen Z. X. Chen C. P. Zhang N. Wang T. X. (2018b). Low-expression of lncRNA FER1L4 Might Be a Prognostic Marker in Osteosarcoma. Eur. Rev. Med. Pharmacol. Sci. 22, 2310–2314. 10.26355/eurrev_201804_14820 29762833

[B10] Cheng C. Zhang Z. Cheng F. Shao Z. (2020). Exosomal lncRNA RAMP2-AS1 Derived from Chondrosarcoma Cells Promotes Angiogenesis through miR-2355-5p/VEGFR2 Axis. Ott 13, 3291–3301. 10.2147/ott.s244652 PMC7182451 32368088

[B11] Cheng S. Zheng J. Liu X. Shi J. Gong F. Zhang X. (2021). Knockdown of 91 H Suppresses the Tumorigenesis of Osteosarcoma via Inducing Methylation of CDK4 Promoter. Technol. Cancer Res. Treat. 20, 1533033821990006. 10.1177/1533033821990006 33499776 PMC7844445

[B12] Cheng Z. Lei Z. Yang P. Si A. Xiang D. Zhou J. (2019). Long Non-coding RNA THOR Promotes Liver Cancer Stem Cells Expansion via β-catenin Pathway. Gene 684, 95–103. 10.1016/j.gene.2018.10.051 30359743

[B13] Di Fiore R. Drago-Ferrante R. Pentimalli F. Di Marzo D. Forte I. M. Carlisi D. (2016). Let-7d miRNA Shows Both Antioncogenic and Oncogenic Functions in Osteosarcoma-Derived 3AB-OS Cancer Stem Cells. J. Cel. Physiol. 231, 1832–1841. 10.1002/jcp.25291 26679758

[B14] Di Fiore R. Drago-Ferrante R. Pentimalli F. Di Marzo D. Forte I. M. D’Anneo A. (2014). MicroRNA-29b-1 Impairs *In Vitro* Cell Proliferation, Self-Renewal and Chemoresistance of Human Osteosarcoma 3AB-OS Cancer Stem Cells. Int. J. Oncol. 45, 2013–2023. 10.3892/ijo.2014.2618 25174983 PMC4432724

[B15] Di Fiore R. Fanale D. Drago-Ferrante R. Chiaradonna F. Giuliano M. De Blasio A. (2013). Genetic and Molecular Characterization of the Human Osteosarcoma 3AB-OS Cancer Stem Cell Line: a Possible Model for Studying Osteosarcoma Origin and Stemness. J. Cel. Physiol. 228, 1189–1201. 10.1002/jcp.24272 23129384

[B16] Fei D. Zhang X. Liu J. Tan L. Xing J. Zhao D. (2018). Long Noncoding RNA FER1L4 Suppresses Tumorigenesis by Regulating the Expression of PTEN Targeting miR-18a-5p in Osteosarcoma. Cel Physiol Biochem 51, 1364–1375. 10.1159/000495554 30481787

[B17] Ferretti V. A. León I. E. (2021). Long Non-coding RNAs in Cisplatin Resistance in Osteosarcoma. Curr. Treat. Options. Oncol. 22, 41. 10.1007/s11864-021-00839-y 33745006

[B18] Fujiwara T. Katsuda T. Hagiwara K. Kosaka N. Yoshioka Y. Takahashi R.-U. (2014). Clinical Relevance and Therapeutic Significance of microRNA-133a Expression Profiles and Functions in Malignant Osteosarcoma-Initiating Cells. Stem Cells 32, 959–973. 10.1002/stem.1618 24715690

[B19] Gao L. Cheng X.-l. Cao H. (2018). LncRNA THOR Attenuates Cisplatin Sensitivity of Nasopharyngeal Carcinoma Cells via Enhancing Cells Stemness. Biochimie 152, 63–72. 10.1016/j.biochi.2018.06.015 29959065

[B20] Gebert L. F. R. Macrae I. J. (2019). Regulation of microRNA Function in Animals. Nat. Rev. Mol. Cel Biol 20, 21–37. 10.1038/s41580-018-0045-7 PMC6546304 30108335

[B21] Ghafouri-Fard S. Shirvani-Farsani Z. Hussen B. M. Taheri M. (2021). The Critical Roles of lncRNAs in the Development of Osteosarcoma. Biomed. Pharmacother. 135, 111217. 10.1016/j.biopha.2021.111217 33433358

[B22] Gill J. Gorlick R. (2021). Advancing Therapy for Osteosarcoma. Nat. Rev. Clin. Oncol. 18 (10), 609–624. 10.1038/s41571-021-00519-8 34131316

[B23] Guo X. Yu L. Zhang Z. Dai G. Gao T. Guo W. (2017). miR-335 Negatively Regulates Osteosarcoma Stem Cell-like Properties by Targeting POU5F1. Cancer Cel Int 17, 29. 10.1186/s12935-017-0398-6 PMC5316195 28239298

[B24] Han Q. Yang J. Yang H. Li C. Li J. Cao Y. (2020). RETRACTED ARTICLE: KIAA1429 Promotes Osteosarcoma Progression by Promoting Stem Cell Properties and Is Regulated by miR-143-3p. Cell Cycle 19, 1172–1185. 10.1080/15384101.2020.1749465 32286148 PMC7217356

[B25] Hattinger C. M. Patrizio M. P. Fantoni L. Casotti C. Riganti C. Serra M. (2021). Drug Resistance in Osteosarcoma: Emerging Biomarkers, Therapeutic Targets and Treatment Strategies. Cancers (Basel) 13, 2878. 10.3390/cancers13122878 34207685 PMC8228414

[B26] Hou Y. Feng H. Jiao J. Qian L. Sun B. Chen P. (2019). Mechanism of miR-143-3p Inhibiting Proliferation, Migration and Invasion of Osteosarcoma Cells by Targeting MAPK7. Artif. Cell Nanomedicine, Biotechnol. 47, 2065–2071. 10.1080/21691401.2019.1620252 31126193

[B27] Huang Y. Han Y. Guo R. Liu H. Li X. Jia L. (2020). Long Non-coding RNA FER1L4 Promotes Osteogenic Differentiation of Human Periodontal Ligament Stromal Cells via miR-874-3p and Vascular Endothelial Growth Factor A. Stem Cel Res Ther 11, 5. 10.1186/s13287-019-1519-z PMC6942378 31900200

[B28] Huang Y. Liu H. Guo R. Han Y. Yang Y. Zhao Y. (2021). Long Non-coding RNA FER1L4 Mediates the Autophagy of Periodontal Ligament Stem Cells under Orthodontic Compressive Force via AKT/FOXO3 Pathway. Front. Cel Dev. Biol. 9, 631181. 10.3389/fcell.2021.631181 PMC7884613 33604341

[B29] Huang Y. Zhang Y. Li X. Liu H. Yang Q. Jia L. (2019). The Long Non-coding RNA Landscape of Periodontal Ligament Stem Cells Subjected to Compressive Force. Eur. J. Orthod. 41, 333–342. 10.1093/ejo/cjy057 30169774

[B30] Humphries B. Wang Z. Yang C. (2021). MicroRNA Regulation of Breast Cancer Stemness. Int. J. Mol. Sci. 22, 3756. 10.3390/ijms22073756 33916548 PMC8038508

[B31] Izadpanah S. Shabani P. Aghebati‐Maleki A. Baghbanzadeh A. Fotouhi A. Bisadi A. (2020). Prospects for the Involvement of Cancer Stem Cells in the Pathogenesis of Osteosarcoma. J. Cel Physiol 235, 4167–4182. 10.1002/jcp.29344 31709547

[B32] Jiang N. Wang X. Xie X. Liao Y. Liu N. Liu J. (2017). lncRNA DANCR Promotes Tumor Progression and Cancer Stemness Features in Osteosarcoma by Upregulating AXL via miR-33a-5p Inhibition. Cancer Lett. 405, 46–55. 10.1016/j.canlet.2017.06.009 28642170

[B33] Kim E. H. Kim J. Y. Kim M. S. Vares G. Ohno T. Takahashi A. (2020). Molecular Mechanisms Underlying the Enhancement of Carbon Ion Beam Radiosensitivity of Osteosarcoma Cells by miR-29b. Am. J. Cancer Res. 10, 4357–4371. 33415004 PMC7783744

[B34] Kong Y. Nie Z. Guo H. Ma C. (2020). LINK-A lncRNA Is Upregulated in Osteosarcoma and Regulates Migration, Invasion and Stemness of Osteosarcoma Cells. Oncol. Lett. 19, 2832–2838. 10.3892/ol.2020.11367 32218837 PMC7068315

[B35] Lei Y. Junxin C. Yongcan H. Xiaoguang L. Binsheng Y. (2020). Role of microRNAs in the Crosstalk between Osteosarcoma Cells and the Tumour Microenvironment. J. Bone Oncol. 25, 100322. 10.1016/j.jbo.2020.100322 33083216 PMC7554654

[B36] Li J. Wang L. Liu Z. Zu C. Xing F. Yang P. (2015). MicroRNA-494 Inhibits Cell Proliferation and Invasion of Chondrosarcoma Cellsin Vivoandin Vitroby Directly Targeting SOX9. Oncotarget 6, 26216–26229. 10.18632/oncotarget.4460 26317788 PMC4694896

[B37] Li M. Ma W. (2021). miR-26a Reverses Multidrug Resistance in Osteosarcoma by Targeting MCL1. Front. Cel Dev. Biol. 9, 645381. 10.3389/fcell.2021.645381 PMC8012539 33816494

[B38] Li P. Y. Wang P. Gao S. G. Dong D. Y. (2020a). Long Noncoding RNA SOX2-OT: Regulations, Functions, and Roles on Mental Illnesses, Cancers, and Diabetic Complications. Biomed. Res. Int. 2020, 2901589. 10.1155/2020/2901589 33294436 PMC7718063

[B39] Li S. Bai H. Chen X. Gong S. Xiao J. Li D. (2020b). Soft Substrate Promotes Osteosarcoma Cell Self-Renewal, Differentiation, and Drug Resistance through miR-29b and its Target Protein Spin 1. ACS Biomater. Sci. Eng. 6, 5588–5598. 10.1021/acsbiomaterials.0c00816 33320589

[B40] Li W. He X. Xue R. Zhang Y. Zhang X. Lu J. (2016a). Combined Over-expression of the Hypoxia-Inducible Factor 2α Gene and its Long Non-coding RNA Predicts Unfavorable Prognosis of Patients with Osteosarcoma. Pathol. - Res. Pract. 212, 861–866. 10.1016/j.prp.2016.06.013 27623205

[B41] Li Y. Liu J.-J. Zhou J.-H. Chen R. Cen C.-Q. (2020c). LncRNA HULC Induces the Progression of Osteosarcoma by Regulating the miR-372-3p/HMGB1 Signalling axis. Mol. Med. 26, 26. 10.1186/s10020-020-00155-5 32188407 PMC7081592

[B42] Li Z. Zhao L. Wang Q. (2016b). Overexpression of Long Non-coding RNA HOTTIP Increases Chemoresistance of Osteosarcoma Cell by Activating the Wnt/β-Catenin Pathway. Am. J. Transl Res. 8, 2385–2393. 27347346 PMC4891451

[B43] Li Z. Li X. Xu D. Chen X. Li S. Zhang L. (2021). An Update on the Roles of Circular RNAs in Osteosarcoma. Cell Prolif 54, e12936. 10.1111/cpr.12936 33103338 PMC7791175

[B44] Li Z. Wang Y. Hu R. Xu R. Xu W. (2018). LncRNA B4GALT1-AS1 Recruits HuR to Promote Osteosarcoma Cells Stemness and Migration via Enhancing YAP Transcriptional Activity. Cel Prolif 51, e12504. 10.1111/cpr.12504 PMC6528912 30182452

[B45] Liang X. Xu C. Wang W. Li X. (2019b). The DNMT1/miR-34a Axis Is Involved in the Stemness of Human Osteosarcoma Cells and Derived Stem-like Cells. Stem Cell Int 2019, 7028901. 10.1155/2019/7028901 PMC6875320 31781245

[B46] Liang X. Xu C. Cao X. Wang W. (2019a). Isovitexin Suppresses Cancer Stemness Property and Induces Apoptosis of Osteosarcoma Cells by Disruption of the DNMT1/miR-34a/Bcl-2 Axis. Cmar 11, 8923–8936. 10.2147/cmar.s222708 PMC6800563 31686915

[B47] Lin J. H. Chen F. N. Wu C. X. Hu S. Q. Ma J. (2020). Long non-coding RNA B4GALT1-Antisense RNA 1/microRNA-30e/SRY-box transcription factor 9 signaling axis contributes to non-small cell lung cancer cell growth. Oncol. Lett. 20, 284. 10.3892/ol.2020.12146 33014162 PMC7520745

[B48] Lin Z. Xie X. Lu S. Liu T. (2021). Noncoding RNAs in Osteosarcoma: Implications for Drug Resistance. Cancer Lett. 504, 91–103. 10.1016/j.canlet.2021.02.007 33587978

[B49] Liu J. Mi B. Wang Y. Shi C. Mi X. Lu Y. (2018). miR-26a Suppresses Osteosarcoma Migration and Invasion by Directly Targeting HMGA1. Oncol. Lett. 15, 8303–8310. 10.3892/ol.2018.8359 29928320 PMC6004719

[B50] Liu K. Ni J. D. Li W. Z. Pan B. Q. Yang Y. T. Xia Q. (2020). The Sp1/FOXC1/HOTTIP/LATS2/YAP/β‐catenin cascade Promotes Malignant and Metastatic Progression of Osteosarcoma. Mol. Oncol. 14, 2678–2695. 10.1002/1878-0261.12760 32634265 PMC7530777

[B51] Liu Z. F. Liang Z. Q. Li L. Zhou Y. B. Wang Z. B. Gu W. F. (2016). MiR-335 Functions as a Tumor Suppressor and Regulates Survivin Expression in Osteosarcoma. Eur. Rev. Med. Pharmacol. Sci. 20, 1251–1257. 27097943

[B52] Lu J. Song G. Tang Q. Yin J. Zou C. Zhao Z. (2017). MiR-26a Inhibits Stem Cell-like Phenotype and Tumor Growth of Osteosarcoma by Targeting Jagged1. Oncogene 36, 231–241. 10.1038/onc.2016.194 27270422

[B53] Lu Y. Li F. Xu T. Sun J. (2016). miRNA-497 Negatively Regulates the Growth and Motility of Chondrosarcoma Cells by Targeting Cdc25A. Oncol. Res. 23, 155–163. 10.3727/096504016x14519157902681 27053344 PMC7838736

[B54] Luo D. J. Li L. J. Huo H. F. Liu X. Q. Cui H. W. Jiang D. M. (2019). MicroRNA-29b Sensitizes Osteosarcoma Cells to Doxorubicin by Targeting Matrix Metalloproteinase 9 (MMP-9) in Osteosarcoma. Eur. Rev. Med. Pharmacol. Sci. 23, 1434–1442. 10.26355/eurrev_201902_17100 30840264

[B55] Ma H. Su R. Feng H. Guo Y. Su G. (2019a). Long Noncoding RNA UCA1 Promotes Osteosarcoma Metastasis through CREB1-Mediated Epithelial-Mesenchymal Transition and Activating PI3K/AKT/mTOR Pathway. J. Bone Oncol. 16, 100228. 10.1016/j.jbo.2019.100228 31011522 PMC6463206

[B56] Ma L. Zhang L. Guo A. Liu L. C. Yu F. Diao N. (2019b). Overexpression of FER1L4 Promotes the Apoptosis and Suppresses Epithelial-Mesenchymal Transition and Stemness Markers via Activating PI3K/AKT Signaling Pathway in Osteosarcoma Cells. Pathol. - Res. Pract. 215, 152412. 10.1016/j.prp.2019.04.004 31000382

[B57] Melendez-Zajgla J. Maldonado V. (2021). The Role of lncRNAs in the Stem Phenotype of Pancreatic Ductal Adenocarcinoma. Int. J. Mol. Sci. 22, 6374. 10.3390/ijms22126374 34203589 PMC8232220

[B58] Palmini G. Marini F. Brandi M. L. (2017). What Is New in the miRNA World Regarding Osteosarcoma and Chondrosarcoma? Molecules 22, 417. 10.3390/molecules22030417 PMC6155266 28272374

[B59] Pan Z. Wu C. Li Y. Li H. An Y. Wang G. (2020). LncRNA DANCR Silence Inhibits SOX5-Medicated Progression and Autophagy in Osteosarcoma via Regulating miR-216a-5p. Biomed. Pharmacother. 122, 109707. 10.1016/j.biopha.2019.109707 31918278

[B60] Pu F.-F. Shi D.-Y. Chen T. Liu Y.-X. Zhong B.-L. Zhang Z.-C. (2021). SP1-induced Long Non-coding RNA SNHG6 Facilitates the Carcinogenesis of Chondrosarcoma through Inhibiting KLF6 by Recruiting EZH2. Cel Death Dis 12, 59. 10.1038/s41419-020-03352-6 PMC7801621 33431838

[B61] Pu F. Chen F. Shao Z. (2016). MicroRNAs as Biomarkers in the Diagnosis and Treatment of Chondrosarcoma. Tumour Biol. 10.1007/s13277-016-5468-1 27730542

[B62] Qu F. Li C.-B. Yuan B.-T. Qi W. Li H.-L. Shen X.-Z. (2016). MicroRNA-26a Induces Osteosarcoma Cell Growth and Metastasis via the Wnt/β-Catenin Pathway. Oncol. Lett. 11, 1592–1596. 10.3892/ol.2015.4073 26893786 PMC4734282

[B63] Rengganaten V. Huang C. J. Tsai P. H. Wang M. L. Yang Y. P. Lan Y. T. (2020). Mapping a Circular RNA-microRNA-mRNA-Signaling Regulatory Axis that Modulates Stemness Properties of Cancer Stem Cell Populations in Colorectal Cancer Spheroid Cells. Int. J. Mol. Sci. 21, 7864. 10.3390/ijms21217864 PMC7672619 33114016

[B64] Schiavone K. Garnier D. Heymann M.-F. Heymann D. (2019). The Heterogeneity of Osteosarcoma: The Role Played by Cancer Stem Cells. Adv. Exp. Med. Biol. 1139, 187–200. 10.1007/978-3-030-14366-4_11 31134502

[B65] Sha S. Yuan D. Liu Y. Han B. Zhong N. (2017). Targeting Long Non-coding RNA DANCR Inhibits Triple Negative Breast Cancer Progression. Biol. Open 6, 1310–1316. 10.1242/bio.023135 28760736 PMC5612229

[B66] Shi P. Li Y. Guo Q. (2021a). Circular RNA circPIP5K1A Contributes to Cancer Stemness of Osteosarcoma by miR-515-5p/YAP axis. J. Transl Med. 19, 464. 10.1186/s12967-021-03124-6 34774083 PMC8590363

[B67] Shi Z. Wang K. Xing Y. Yang X. (2021b). CircNRIP1 Encapsulated by Bone Marrow Mesenchymal Stem Cell-Derived Extracellular Vesicles Aggravates Osteosarcoma by Modulating the miR-532-3p/AKT3/PI3K/AKT Axis. Front. Oncol. 11, 658139. 10.3389/fonc.2021.658139 34660257 PMC8511523

[B68] Shui X. Zhou C. Lin W. Yu Y. Feng Y. Kong J. (2017). Long Non-coding RNA BCAR4 Promotes Chondrosarcoma Cell Proliferation and Migration through Activation of mTOR Signaling Pathway. Exp. Biol. Med. (Maywood) 242, 1044–1050. 10.1177/1535370217700735 28399646 PMC5444642

[B69] Siegel R. L. Miller K. D. Fuchs H. E. Jemal A. (2021). Cancer Statistics, 2021. CA A. Cancer J. Clin. 71, 7–33. 10.3322/caac.21654 33433946

[B70] Slaby O. (2016). Non-coding RNAs as Biomarkers for Colorectal Cancer Screening and Early Detection. Adv. Exp. Med. Biol. 937, 153–170. 10.1007/978-3-319-42059-2_8 27573899

[B71] Slack F. J. Chinnaiyan A. M. (2019). The Role of Non-coding RNAs in Oncology. Cell 179, 1033–1055. 10.1016/j.cell.2019.10.017 31730848 PMC7347159

[B72] Song H. Xu Y. Shi L. Xu T. Fan R. Cao M. (2018). LncRNA THOR Increases the Stemness of Gastric Cancer Cells via Enhancing SOX9 mRNA Stability. Biomed. Pharmacother. 108, 338–346. 10.1016/j.biopha.2018.09.057 30227327

[B73] Song Q.-C. Shi Z.-B. Zhang Y.-T. Ji L. Wang K.-Z. Duan D.-P. (2014). Downregulation of microRNA-26a Is Associated with Metastatic Potential and the Poor Prognosis of Osteosarcoma Patients. Oncol. Rep. 31, 1263–1270. 10.3892/or.2014.2989 24452597

[B74] Sui Y. Han Y. Zhao X. Li D. Li G. (2018). Long Non-coding RNA GClnc1 Promotes Tumorigenesis in Osteosarcoma by Inhibiting P53 Signaling. Biochem. Biophysical Res. Commun. 507, 36–42. 10.1016/j.bbrc.2018.10.135 30454890

[B75] Sun J. Wang X. Fu C. Wang X. Zou J. Hua H. (2016). Long Noncoding RNA FGFR3-AS1 Promotes Osteosarcoma Growth through Regulating its Natural Antisense Transcript FGFR3. Mol. Biol. Rep. 43, 427–436. 10.1007/s11033-016-3975-1 27022737

[B76] Sun X. Dai G. Yu L. Hu Q. Chen J. Guo W. (2018). miR-143-3p Inhibits the Proliferation, Migration and Invasion in Osteosarcoma by Targeting FOSL2. Sci. Rep. 8, 606. 10.1038/s41598-017-18739-3 29330462 PMC5766605

[B77] Sung H. Ferlay J. Siegel R. L. Laversanne M. Soerjomataram I. Jemal A. (2021). Global Cancer Statistics 2020: GLOBOCAN Estimates of Incidence and Mortality Worldwide for 36 Cancers in 185 Countries. CA A. Cancer J. Clin. 71, 209–249. 10.3322/caac.21660 33538338

[B78] Tan X. Fan S. Wu W. Zhang Y. (2015). MicroRNA-26a Inhibits Osteosarcoma Cell Proliferation by Targeting IGF-1. Bone Res. 3, 15033. 10.1038/boneres.2015.33 27468358 PMC4948281

[B79] Tang Y. Ji F. (2019). lncRNA HOTTIP Facilitates Osteosarcoma Cell Migration, Invasion and Epithelial-Mesenchymal Transition by Forming a Positive Feedback Loop with C-Myc. Oncol. Lett. 18, 1649–1656. 10.3892/ol.2019.10463 31423232 PMC6607149

[B80] Vares G. Ahire V. Sunada S. Ho Kim E. Sai S. Chevalier F. (2020). A Multimodal Treatment of Carbon Ions Irradiation, miRNA-34 and mTOR Inhibitor Specifically Control High-Grade Chondrosarcoma Cancer Stem Cells. Radiother. Oncol. 150, 253–261. 10.1016/j.radonc.2020.07.034 32717360

[B81] Wang B. Ye Q. Zou C. (2020a). Long Non-coding RNA THOR Enhances the Stem Cell-like Traits of Triple-Negative Breast Cancer Cells through Activating β-Catenin Signaling. Med. Sci. Monit. 26, e923507. 10.12659/MSM.923507 32665537 PMC7366791

[B82] Wang L. Wang P. Su X. Zhao B. (2020b). Circ_0001658 Promotes the Proliferation and Metastasis of Osteosarcoma Cells via Regulating miR‐382‐5p/YB‐1 axis. Cell Biochem Funct 38, 77–86. 10.1002/cbf.3452 31758574

[B83] Wang T. Wang D. Zhang L. Yang P. Wang J. Liu Q. (2019). The TGFβ-miR-499a-SHKBP1 Pathway Induces Resistance to EGFR Inhibitors in Osteosarcoma Cancer Stem Cell-like Cells. J. Exp. Clin. Cancer Res. 38, 226. 10.1186/s13046-019-1195-y 31138318 PMC6540516

[B84] Wang Y. Wang N. Zeng X. Sun J. Wang G. Xu H. (2017a). MicroRNA-335 and its Target Rock1 Synergistically Influence Tumor Progression and Prognosis in Osteosarcoma. Oncol. Lett. 13, 3057–3065. 10.3892/ol.2017.5818 28521412 PMC5431301

[B85] Wang Y. Yang T. Zhang Z. Lu M. Zhao W. Zeng X. (2017b). Long Non-coding RNA TUG1 Promotes Migration and Invasion by Acting as a ceRNA of miR-335-5p in Osteosarcoma Cells. Cancer Sci. 108, 859–867. 10.1111/cas.13201 28205334 PMC5448616

[B86] Wang Y. Yao J. Meng H. Yu Z. Wang Z. Yuan X. (2015). A Novel Long Non-coding RNA, Hypoxia-Inducible Factor-2α Promoter Upstream Transcript, Functions as an Inhibitor of Osteosarcoma Stem Cells *In Vitro* . Mol. Med. Rep. 11, 2534–2540. 10.3892/mmr.2014.3024 25434862 PMC4337490

[B87] Wang Y. Zeng X. Wang N. Zhao W. Zhang X. Teng S. (2018). Long Noncoding RNA DANCR, Working as a Competitive Endogenous RNA, Promotes ROCK1-Mediated Proliferation and Metastasis via Decoying of miR-335-5p and miR-1972 in Osteosarcoma. Mol. Cancer 17, 89. 10.1186/s12943-018-0837-6 29753317 PMC5948795

[B88] Wang Y. Zhang Y. Yang T. Zhao W. Wang N. Li P. (2017c). Long Non-coding RNA MALAT1 for Promoting Metastasis and Proliferation by Acting as a ceRNA of miR-144-3p in Osteosarcoma Cells. Oncotarget 8, 59417–59434. 10.18632/oncotarget.19727 28938647 PMC5601743

[B89] Wang Y. Zhao W. Fu Q. (2013). miR-335 Suppresses Migration and Invasion by Targeting ROCK1 in Osteosarcoma Cells. Mol. Cel Biochem 384, 105–111. 10.1007/s11010-013-1786-4 23975506

[B90] Wang Z. Tan M. Chen G. Li Z. Lu X. (2017d). LncRNA SOX2-OT Is a Novel Prognostic Biomarker for Osteosarcoma Patients and Regulates Osteosarcoma Cells Proliferation and Motility through Modulating SOX2. IUBMB Life 69, 867–876. 10.1002/iub.1681 28960757

[B91] Wei Q. F. Yao J. S. Yang Y. T. (2019). MicroRNA-1247 Inhibits the Viability and Metastasis of Osteosarcoma Cells via Targeting NRP1 and Mediating Wnt/β-Catenin Pathway. Eur. Rev. Med. Pharmacol. Sci. 23, 7266–7274. 10.26355/eurrev_201909_18831 31539113

[B92] Wu D.-M. Zheng Z.-H. Zhang Y.-B. Fan S.-H. Zhang Z.-F. Wang Y.-J. (2019a). Down-regulated lncRNA DLX6-AS1 Inhibits Tumorigenesis through STAT3 Signaling Pathway by Suppressing CADM1 Promoter Methylation in Liver Cancer Stem Cells. J. Exp. Clin. Cancer Res. 38, 237. 10.1186/s13046-019-1239-3 31171015 PMC6554918

[B93] Wu H. He Y. Chen H. Liu Y. Wei B. Chen G. (2019b). Lnc RNA THOR Increases Osteosarcoma Cell Stemness and Migration by Enhancing SOX 9 mRNA Stability. FEBS Open Bio 9, 781–790. 10.1002/2211-5463.12620 PMC6443997 30984551

[B94] Xie Y. Deng H. Wei R. Sun W. Qi Y. Yao S. (2019). Overexpression of miR-335 Inhibits the Migration and Invasion of Osteosarcoma by Targeting SNIP1. Int. J. Biol. Macromolecules 133, 137–147. 10.1016/j.ijbiomac.2019.04.016 30954590

[B95] Xu M. Jin H. Xu C.-X. Sun B. Mao Z. Bi W.-Z. (2014). miR-382 Inhibits Tumor Growth and Enhance Chemosensitivity in Osteosarcoma. Oncotarget 5, 9472–9483. 10.18632/oncotarget.2418 25344865 PMC4253447

[B96] Xu M. Jin H. Xu C.-X. Sun B. Song Z.-G. Bi W.-Z. (2015). miR-382 Inhibits Osteosarcoma Metastasis and Relapse by Targeting Y Box-Binding Protein 1. Mol. Ther. 23, 89–98. 10.1038/mt.2014.197 25292190 PMC4426807

[B97] Xu W. Li Z. Zhu X. Xu R. Xu Y. (2018). miR-29 Family Inhibits Resistance to Methotrexate and Promotes Cell Apoptosis by Targeting COL3A1 and MCL1 in Osteosarcoma. Med. Sci. Monit. 24, 8812–8821. 10.12659/msm.911972 30518744 PMC6292150

[B98] Yan G.-N. Lv Y.-F. Guo Q.-N. (2016). Advances in Osteosarcoma Stem Cell Research and Opportunities for Novel Therapeutic Targets. Cancer Lett. 370, 268–274. 10.1016/j.canlet.2015.11.003 26571463

[B99] Yan J.-P. Xiang R.-M. (2021). Effect Assessment of Methotrexate in Combination with Other Chemotherapeutic Agents for Osteosarcoma in Children. Medicine (Baltimore) 100, e25534. 10.1097/md.0000000000025534 34011024 PMC8137068

[B100] Yang G. Wu Y. Wan R. Sang H. Liu H. Huang W. (2021). The Role of Non-coding RNAs in the R-egulation, D-iagnosis, P-rognosis and T-reatment of O-steosarcoma (Review). Int. J. Oncol. 59, 69. 10.3892/ijo.2021.5249 34296296

[B101] Yang L. Li H. Huang A. (2020). MiR-429 and MiR-143-3p Function as Diagnostic and Prognostic Markers for Osteosarcoma. Clin. Lab. 66. 10.7754/Clin.Lab.2020.191237 33073957

[B102] Yao J. Geng P. Li Y. Chen H. Li J. Zhu Y. (2015). Knockdown of a HIF-2α Promoter Upstream Long Noncoding RNA Impairs Colorectal Cancer Stem Cell Properties *In Vitro* through HIF-2α Downregulation. Ott 8, 3467–3474. 10.2147/ott.s81393 PMC4664519 26648739

[B103] Yao X.-Y. Liu J.-F. Luo Y. Xu X.-Z. Bu J. (2021). LncRNA HOTTIP Facilitates Cell Proliferation, Invasion, and Migration in Osteosarcoma by Interaction with PTBP1 to Promote KHSRP Level. Cell Cycle 20, 283–297. 10.1080/15384101.2020.1870820 33475442 PMC7889103

[B104] Ye F. Tian L. Zhou Q. Feng D. (2019). LncRNA FER1L4 Induces Apoptosis and Suppresses EMT and the Activation of PI3K/AKT Pathway in Osteosarcoma Cells via Inhibiting miR-18a-5p to Promote SOCS5. Gene 721, 144093. 10.1016/j.gene.2019.144093 31473323

[B105] Yu J. E. Ju J. A. Musacchio N. Mathias T. J. Vitolo M. I. (2020). Long Noncoding RNA DANCR Activates Wnt/β-Catenin Signaling through MiR-216a Inhibition in Non-small Cell Lung Cancer. Biomolecules 10, 1646. 10.3390/biom10121646 PMC7764320 33302540

[B106] Yu Y. Wang L. Li Z. Zheng Y. Shi Z. Wang G. (2021). Long Noncoding RNA CRNDE Functions as a Diagnostic and Prognostic Biomarker in Osteosarcoma, as Well as Promotes its Progression via Inhibition of miR-335-3p. J. Biochem. Mol. Toxicol. 35, e22734. 10.1002/jbt.22734 33522065

[B107] Yuan S.-x. Wang J. Yang F. Tao Q.-f. Zhang J. Wang L.-l. (2016). Long Noncoding RNADANCRincreases Stemness Features of Hepatocellular Carcinoma by Derepression ofCTNNB1. Hepatology 63, 499–511. 10.1002/hep.27893 25964079

[B108] Zhang K. Zhang C. Liu L. Zhou J. (2014). A Key Role of microRNA-29b in Suppression of Osteosarcoma Cell Proliferation and Migration via Modulation of VEGF. Int. J. Clin. Exp. Pathol. 7, 5701–5708. 25337211 PMC4203182

[B109] Zhang P.-r. Ren J. Wan J.-s. Sun R. Li Y. (2020a). Circular RNA Hsa_circ_0002052 Promotes Osteosarcoma via Modulating miR-382/STX6 axis. Hum. Cel 33, 810–818. 10.1007/s13577-020-00335-9 32274658

[B110] Zhang P. Li J. Song Y. Wang X. (2017). MiR-129-5p Inhibits Proliferation and Invasion of Chondrosarcoma Cells by Regulating SOX4/Wnt/β-Catenin Signaling Pathway. Cel Physiol Biochem 42, 242–253. 10.1159/000477323 28535514

[B111] Zhang W. Li J. Z. Tai Q. Y. Tang J. J. Huang Y. H. Gao S. B. (2020b). LncRNA DANCR Regulates Osteosarcoma Migration and Invasion by Targeting miR-149/MSI2 axis. Eur. Rev. Med. Pharmacol. Sci. 24, 6551–6560. 10.26355/eurrev_202006_21639 32633342

[B112] Zhang Y. Pan Y. Xie C. Zhang Y. (2018). miR-34a Exerts as a Key Regulator in the Dedifferentiation of Osteosarcoma via PAI-1-Sox2 axis. Cel Death Dis 9, 777. 10.1038/s41419-018-0778-4 PMC6039486 29991717

[B113] Zhang Z. Wu X. Han Q. Huang Z. (2021). Downregulation of Long Non-coding RNA UCA1 Represses Tumorigenesis and Metastasis of Osteosarcoma via miR-513b-5p/E2F5 axis. Anticancer Drugs 32, 602–613. 10.1097/cad.0000000000001034 33595944

[B114] Zhao B. Liu K. Cai L. (2019a). LINK-A lncRNA Functions in the Metastasis of Osteosarcoma by Upregulating HIF1α. Oncol. Lett. 17, 5005–5011. 10.3892/ol.2019.10177 31186711 PMC6507337

[B115] Zhao D. Wang S. Chu X. Han D. (2019b). LncRNA HIF2PUT Inhibited Osteosarcoma Stem Cells Proliferation, Migration and Invasion by Regulating HIF2 Expression. Artif. Cell Nanomedicine, Biotechnol. 47, 1342–1348. 10.1080/21691401.2019.1596934 30966832

[B116] Zhao F. Lv J. Gan H. Li Y. Wang R. Zhang H. (2015). MiRNA Profile of Osteosarcoma with CD117 and Stro-1 Expression: miR-1247 Functions as an Onco-miRNA by Targeting MAP3K9. Int. J. Clin. Exp. Pathol. 8, 1451–1458. 25973030 PMC4396303

[B117] Zhou J. Wu S. Chen Y. Zhao J. Zhang K. Wang J. (2015). microRNA-143 Is Associated with the Survival of ALDH1+CD133+ Osteosarcoma Cells and the Chemoresistance of Osteosarcoma. Exp. Biol. Med. (Maywood) 240, 867–875. 10.1177/1535370214563893 25576341 PMC4935406

[B118] Zhu K. Liu L. Zhang J. Wang Y. Liang H. Fan G. (2016). MiR-29b Suppresses the Proliferation and Migration of Osteosarcoma Cells by Targeting CDK6. Protein Cell 7, 434–444. 10.1007/s13238-016-0277-2 27230400 PMC4887333

[B119] Zini R. Rossi C. Norfo R. Pennucci V. Barbieri G. Ruberti S. (2016). miR-382-5p Controls Hematopoietic Stem Cell Differentiation through the Downregulation of MXD1. Stem Cell Development 25, 1433–1443. 10.1089/scd.2016.0150 27520398

[B120] Zou Y. Huang Y. Yang J. Wu J. Luo C. (2017). miR-34a Is Downregulated in Human Osteosarcoma Stem-like Cells and Promotes Invasion, Tumorigenic Ability and Self-Renewal Capacity. Mol. Med. Rep. 15, 1631–1637. 10.3892/mmr.2017.6187 28260055 PMC5364984

